# Temperature Oscillation Modulated Self-Assembly of Periodic Concentric Layered Magnesium Carbonate Microparticles

**DOI:** 10.1371/journal.pone.0088648

**Published:** 2014-02-10

**Authors:** Shihong Li, Zheng Jim Wang, Ting-Tung Chang

**Affiliations:** Small Animal Imaging Facility, Laboratory of Translational Imaging, Van Andel Research Institute, Grand Rapids, Michigan, United States of America; Brandeis University, United States of America

## Abstract

Intriguing patterns of periodic, concentric, layered, mineral microstructure are present in nature and organisms, yet they have elusive geneses. We hypothesize temperature oscillation can be an independent factor that causes the self-assembly of such patterns in mineral phases synthesized in solution. Static experiments verify that rhythmic concentric multi-layered magnesium carbonate microhemispheres can be synthesized from bicarbonate solution by temperature oscillation, without use of a chemical template, additive or gel-diffusion system. Appropriate reactant concentration and initial pH value can restrain the competitive growth of other mineral generations. Polarized light microscopy images indicate the microhemispheres are crystalline and the crystallinity increases with incubation time. The thickness of a single mineral layer of microhemisphere in microscale is precisely controlled by the waveform parameters of the temperature oscillation, while the layer number, which can reach tens to about one hundred, is constrained by the temperature oscillation period number. FT-IR spectra show that these microhemispheres synthesized under different conditions can be identified as the basic form of magnesium carbonate, hydromagnesite (Mg_5_(CO_3_)_4_(OH)_2_⋅4H_2_O). SEM images exhibit the characteristic microscopic texture of the alternating dark and light rings of these microhemispheres. TEM images and ED patterns suggest the nanoflakes of microhemispheres are present in polycrystalline form with some degree of oriented assembly. The temperature oscillation modulated self-assembly may offer a new mechanism to understand the formation of layered microstructure of minerals in solution, and provide a non-invasive and programmable means to synthesize hierarchically ordered materials.

## Introduction

Self-assembly is a fundamental aspect of nature and organisms to form organized structures [1]–[3]. The diverse, intriguing patterns of periodic concentric layered microstructure are present in natural materials, such as ooids [4]–[8]; biological samples from living organisms, such as gallstones, otolith, Liesegang ring-like structure in certain rare pathologic zone and yolk spherocrystal [9]–[13]; and laboratory products, such as the Liesegang rings formed in gel-diffusion systems [14], [15]. Such patterns often have elusive formation mechanisms. For example, the origin of ooids has long been postulated to be influenced by either biotic or abiotic factors, in addition to the role of organic matter [4]–[8]. From the view of classic ion-by-ion attachment crystallization theory, it is unlikely that such patterned structures can be self-assembled by the mineral phase synthesized in aqueous solution without the participation of another chemical component as mediator. However, the non-classic nucleation and crystallization theory developed in recent years [3], [16]–[24] allows for early structural preformation, and provides the possibility to form complex off-equilibrium crystal structures. Stable nanoscopic clusters of metal oxides, oxyhydroxides and carbonates can be formed in the aqueous suspensions [17]–[20], [25]. The chemical mediation method, involving a chemical template, additive or gel-diffusion system [3], [17], [26] has already been extensively applied to the synthesis of several complex, off-equilibrium structures. Interestingly, short term temperature fluctuations can induce dramatic structural marks onto the otoliths of some incubating fishes, such as salmonoids, and the otolith thermal marking technique has been widely used to mark juvenile fish for their origin identification [10], [11].

Temperature is a key thermodynamic factor determining material state and transformation. Its important role has been embodied in the syntheses of polymorphic carbonate mineral. The polymorph discriminations of both calcium carbonate and magnesium-amorphous calcium carbonate have been realized by simply tuning the reaction temperature without using any organic additives [27]–[30]. The temperature modulated formation of different polymorphs of calcium carbonate in double-jet experiments was observed in coincidence with the crystal energy sequence of different mineral phase [28]. The influence of temperature on the preferential chemical forms, phase transformation and morphology discrimination of magnesium carbonate has been extensively studied [31]–[37]. The hydrated amorphous carbonates are metastable phase and precursors, providing a low energy pathway for carbonate mineralization to different crystal forms following energetically downhill sequence [38]–[39]. With these hints, the varying temperature may favor the non-classic nucleation of minerals and may have an exclusive constraint on this nucleation and subsequent mineral intermediate formation.

We hypothesize that temperature oscillation can be an independent factor causing the self-assembly of periodic patterned microstructure of minerals, if the mineralization process is dominated by the non-classic nucleation of nanoscopic clusters and the heterogeneously nucleated intermediates have a structural preformation property [3], [17], [20]–[22]. Magnesium is an abundant element dissolved in seawater and present in marine ooids [5], [8]. It also plays an important role in biomineralization [26], [40]. Magnesium carbonate hydrates, present in different hydrated and basic forms including nesquehonite (MgCO_3_⋅3H_2_O), lansfordite (MgCO_3_⋅5H_2_O), hydromagnesite (Mg_5_(CO_3_)_4_(OH)_2_⋅4H_2_O) and artinite (Mg_2_CO_3_(OH)_2_⋅3H_2_O), are of significance in sedimentary geology and planetology [41], [42], and extremely important to the geo-sequestration of atmospheric CO_2_ [43], [44]. They have also been widely used for various industrial applications including pharmaceuticals and cosmetic manufacturing [45], [46]. Recent studies have investigated the formation of nanoclusters of magnesium carbonate with different sizes in the pre-nucleation stage, which may be crucial for the mineral crystallization process [47], [48]. Herein, we report that temperature oscillation can modulate the self-assembled synthesis of rhythmic concentric multi-layered microhemispheres of magnesium carbonate.

## Materials and Methods

### Incubation of magnesium bicarbonate solutions under thermostatic and temperature oscillation conditions

ACS grade chemicals and high purity water (≥ 18.2 MΩ·cm) were used for the chemical experiment. Aqueous solutions of magnesium bicarbonate were prepared by mixing 120–240 mM magnesium acetate buffers (MgAc_2_), pH 6.0–8.2 or 240 mM magnesium chloride (MgCl_2_) with 200–500 mM sodium bicarbonate buffers (NaHCO_3_) in 200 µl clear polypropylene microcentrifuge tubes with an attached cap at room temperature. The sample volumes were 50–90 percent of tube capacity. The final concentrations of Mg^2+^ and HCO_3_^−^ were in the range of 40–137 mM and 67–250 mM, respectively; the molar ratios of Mg^2+^/HCO_3_^−^ varied from 0.5 to 1.2. Moreover, higher concentrations of MgCl_2_ and NaHCO_3_, and NaHCO_3_ adjusted with 0035 M sodium hydroxide to different pH values up to 10.7 were also used for magnesium bicarbonate solution preparation to test influence of concentration and pH value on mineralization generation. Magnesium sulfate solution (MgSO_4_) was also used as an alternative magnesium source for magnesium bicarbonate solution preparation for a few synthesis experiments of magnesium carbonate.

Following mixing, the magnesium bicarbonate samples were incubated undisturbed under thermostatic conditions in a precise incubator or under oscillatory temperature conditions that were produced passively in a water bath with apparent constant temperature (*T*) settings (35–70°C). The sample temperature was monitored with a type K thermometer equipped with a micro thermocouple (Extech 421501). The representative sample tubes of a reaction system were observed with naked eyes and optical microscope at different times to track the mineralization process. Many samples were also incubated in a programmable PCR thermocycler (DNA Engine Tetrad 2 thermal cycler, Bio-Rad), while the lid temperature was set to 100°C to prevent vapor condensation. The pH values of the mixtures freshly prepared and after incubation were measured using a HI 2221 pH meter with a HI 1083 micro pH electrode (Hanna Instruments).

### Optical microscopic observation

The magnesium carbonate microparticles synthesized in the magnesium bicarbonate solutions under different conditions were observed in-situ or after loading onto slides by an Olympus MVX10 stereo microscope or a Nikon E600 microscope using either transmitted light mode or reflected light mode. The fragments of microhemispheres created by mechanical crushing or cutting were also observed on slides. The microhemispheres and other mineral generations were also observed by polarized light microscopy, which was realized with the Olympus MVX10 stereo microscope by inserting two circular polarizers on the optical path, one was beneath the specimen stage and the other was above the objective. The digital images were recorded.

### Fourier Transform infrared spectroscopy (FT-IR)

The synthesized magnesium carbonate microparticles were gently washed with water 3 times, dried at 35°C in air, and then ground into powder. The IR spectra of the powder samples were recorded with a JASCO FT/IR-4100typeA spectrometer in the attenuated total reflection mode in the range of 4000–600 cm^−1^. The resolution was 4 cm^−1^ and 32 scans were signal-averaged in each interferogram. The background scan was recorded before the sample measurement and thereafter subtracted from the spectrum for the sample.

### Scanning electron microscope (SEM) observation

The air dried magnesium carbonate microparticles and their crushed fragments were loaded on high vacuum carbon tabs and observed with a JEOL 6610LV scanning electron microscope. Then the morphology of microparticles was observed at different magnifications and the digital images were recorded.

### Transmission electron microscope (TEM) observation with electron diffraction (ED) pattern analysis

For TEM observation, the synthesized magnesium carbonate microparticles were gently washed with water 3 times, then dried at room temperature in air for no more than one day. The dried samples were ground with plastic tip, suspended in pure ethanol and ultrasonicated for 5 minutes, then a drop was transformed onto a carbon coated Cu TEM grid. The grid was thoroughly dried at room temperature before observation with TEM. TEM images and ED patterns were acquired with a JEOL 2200FS 200 kV field emission transmission electron microscope.

## Results and Discussion

**Figure 1:**
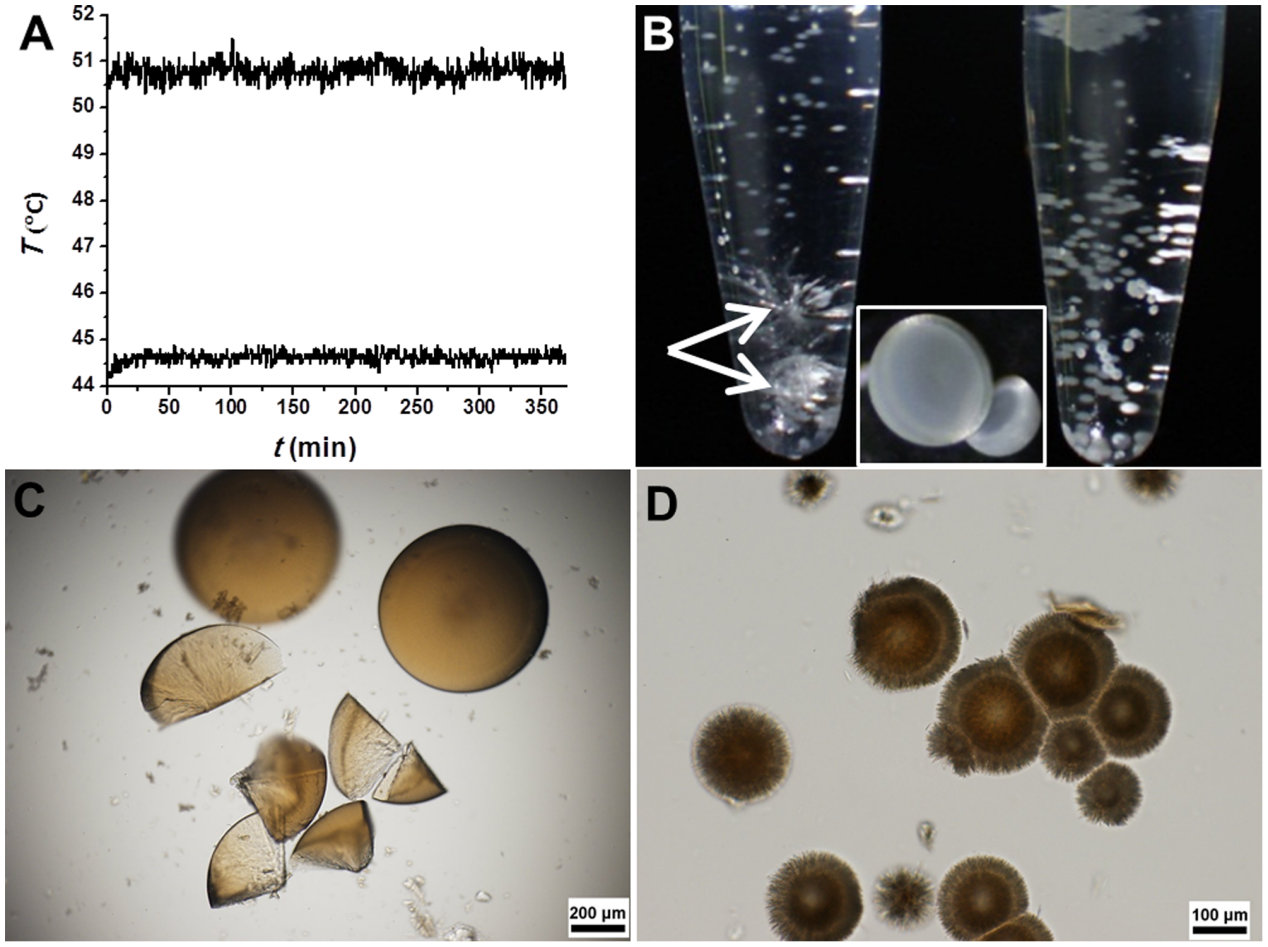
Magnesium carbonate microparticles synthesized under thermostatic conditions. (A) Representative temperature vs. time curves. (B) Reflected light microscopic stereo image of reaction tubes containing microhemispheres with rod-like crystals (arrow) or without. The insert shows the shape of microhemispheres. (C and D) Transmitted light microscopic image of wet microhemispheres. (C) Microhemispheres and fragments having non-layered internal structure. The sample was from a mixture of 240 mM MgCl_2_ with 500 mM NaHCO_3_ (V/V, 1∶1) incubated at 50.8±0.2°C for 14 h. (D) Microhemispheres with a nearly homogeneous core structure and a radially aligned fibrous shell. The sample was from a mixture of 240 mM MgAc_2_, pH 7 with 200 mM NaHCO_3_ (V/V, 1∶2) incubated at 67.4±0.3°C for 14 h.

In the control experiment, the magnesium bicarbonate solutions were heated under thermostatic conditions (representative temperature-time curves shown in Fig. 1A). Firstly, the influence of reactant concentration on mineralization was investigated. It was found that high reactant concentration tend to produce numerous amorphous matter as an initial mineralization stage, such as, when the concentration of Mg(HCO_3_)_2_ solution prepared by mixing of MgCl_2_ and NaHCO_3_ reached 300 mM, the clear solution turned to a cloudy suspension in a few minutes at room temperature. This cloudy suspension was affirmed to be amorphous phase by dark view under cross-polarized light microscopy observation. The amorphous phase produced in these high concentration mineralization systems could experience complicated transformation during static heating at different temperatures. It could transform into clustered rod-like microcrystals and microhemispheres at the interface of the solution and tube wall in a few hours or longer time of incubation (Fig. S1). Though microhemispheres could be formed without concurrence of rod-like microcrystals in some reactant tubes, these high concentration mineralization systems often generated rod-like microcrystals. The coexistence of competitive crystallization pathways in these high concentration mineralization systems reflected the heterogeneous nucleation from amorphous precursor, which probably had low energy difference or was affected by some kinetic factors. The appearance and enlargement of microhemispheres companying with the reduction of clustered rod-like microcrystals was also observed in some reaction tubes at temperature roughly above 55°C, in accord with the literature reported crystalline transformation of magnesium carbonate [34], [35]. An increase of crystallinity of microhemispheres was also observed with incubation time by the brightness and hues of polarized light signal under cross-polarized light microscopy (Fig. S1), reflecting the influence of Ostwald ripening on crystallization of microhemispheres.

Magnesium bicarbonate solutions having high initial pH values prepared with NaHCO_3_ adjusted with concentrated NaOH also tended to form amorphous mineral matter. Such as, cloudy phenomenon appeared instantly or in a few minutes after mixing 120 mM MgCl_2_ with 300 mM NaHCO_3_, pH 10.0–10.7 (V/V 1∶1). Optical imaging showed that static heating of these mineralization systems of high initial pH induced gradual crystallization within the colloidal suspension and resulted in the formation of massive tiny sphere-shaped crystalline microparticles and a small quantity of bigger but relatively loose sphere-shaped microparticles with a hard core or not (Fig. S2).

Under other experimental conditions mentioned in the experimental method section, the magnesium bicarbonate solutions either gradually turned slightly cloudy after tens of minutes of incubation by the formation of amorphous mineral precursors under conditions of high temperature or high reactant concentration, or showed no noticeable cloudiness. The colloidal amorphous precursors were unstable and tended to either quickly disappear or else aggregate to form some irregular microparticles. Then, over several hours to several days of incubation at different temperatures, clustered rod-like microcrystals or microhemispheres of magnesium carbonate appeared and grew slowly at the interface of the solution and tube wall (Fig. 1B). These microhemispheres had relatively homogeneous internal structure with or without a loose shell composed of radially aligned microfibers under optical microscopy observation (Fig. 1C and 1D).

**Figure 2:**
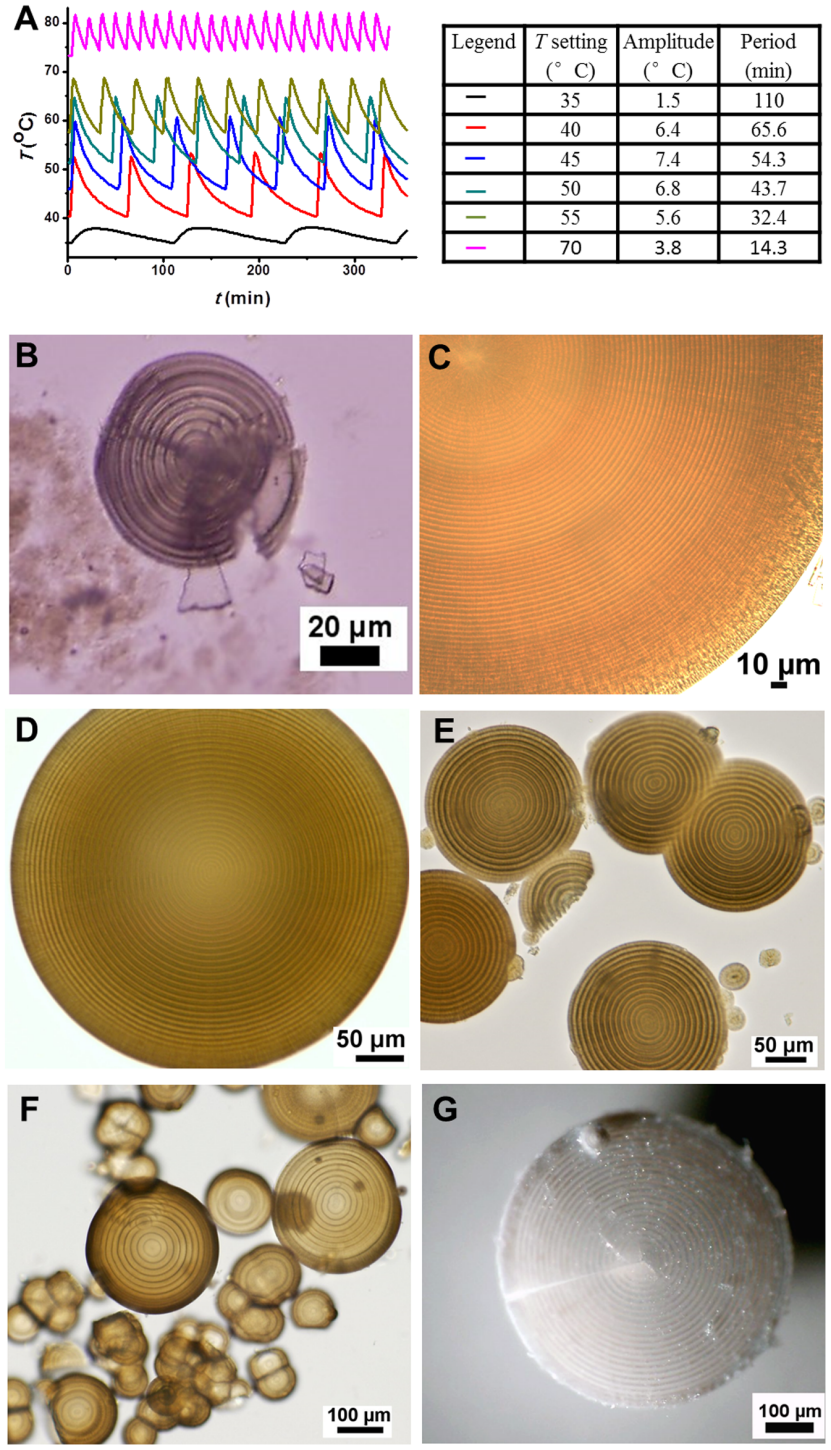
Magnesium carbonate microhemispheres synthesized under passively produced temperature oscillation conditions. (A) Temperature oscillation patterns at different *T* settings. (B–G) Typical optical images. (B) A crushed microhemisphere synthesized from a mixture of 120 mM MgAc_2_, pH 6.5 and 200 mM NaHCO_3_ (1∶1, v/v) at *T* setting of 55°C for 14 h showing dissociated single layer. (C) Partial of a microhemisphere synthesized from a mixture of 120 mM MgAc_2_, pH 6.5 and 200 mM NaHCO_3_ (1∶1, v/v) at *T* setting of 35°C. Layer thickness (LT): 3.3±0.1 µm. (D) and (E) Microhemispheres synthesized from a mixture of 120 mM MgAc_2_, pH 6.0 and 200 mM NaHCO_3_ (1∶1, v/v). (D) at *T* setting of 40°C, LT: 6.4±0.3 µm, (E) at *T* setting of 50°C, LT: 6.9±0.3 µm. (F) and (G) Microspheres synthesized from a mixture of 240 mM MgCl_2_ and 500 mM NaHCO_3_ (1∶1, v/v). (F) at *T* setting of 45°C, LT: 12.8±0.9 µm, (G) at *T* setting of 55°C, LT: 13.0±0.4 µm.

**Figure 3:**
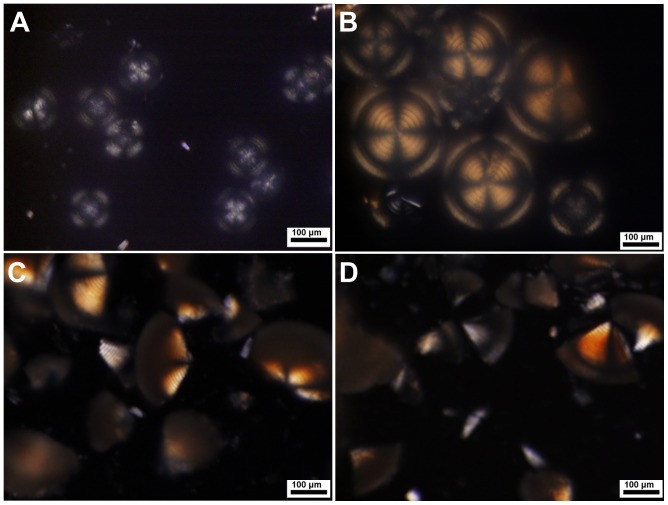
Polarized light images of magnesium carbonate microhemispheres synthesized under temperature oscillation conditions. (A) Microhemispheres synthesized from a mixture of 240 mM MgAc_2_, pH 6.5 with 400 mM NaHCO_3_ (V/V, 1∶1) at *T* setting of 55°C for 6 h. (B) Microhemispheres synthesized from a mixture of 240 mM MgCl_2_ with 500 mM NaHCO_3_ (V/V, 1∶1) at *T* setting of 55°C for 6 h. (C) and (D) Fragments of microhemispheres synthesized from a mixture same as that of (B) at *T* setting of 55°C for 14 h and *T* setting of 50°C for 14 h, respectively.

**Figure 4:**
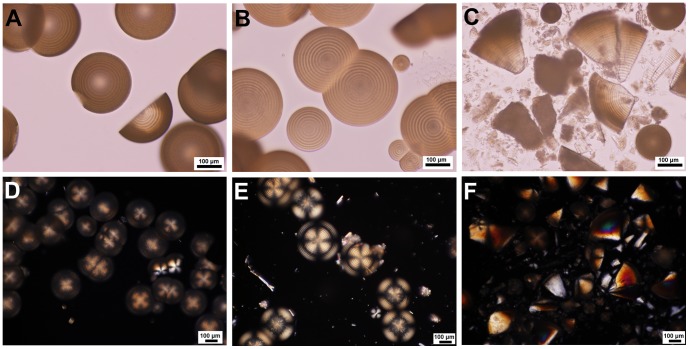
Optical microscopic images of magnesium carbonate microhemispheres synthesized from high concentration of magnesium bicarbonate under temperature oscillation condition. Mg(HCO_3_)_2_ solutions were prepared by mixing of MgCl_2_ and NaHCO_3_ (Mg^2+^/HCO_3_^2−^ molar ratio, 2∶1). The upper images were transmitted light images. (A) 200 mM Mg(HCO_3_)_2_ was incubated at *T* setting of 55°C for 6.5 h. (B) and (C) 333 mM Mg(HCO_3_)_2_ was incubated at *T* setting of 55°C for 6.5 h and 22 h, respectively. The lower images, (D), (E) and (F) were cross-polarized light images of samples from same systems as (A), (B) and (C), respectively.

**Figure 5:**
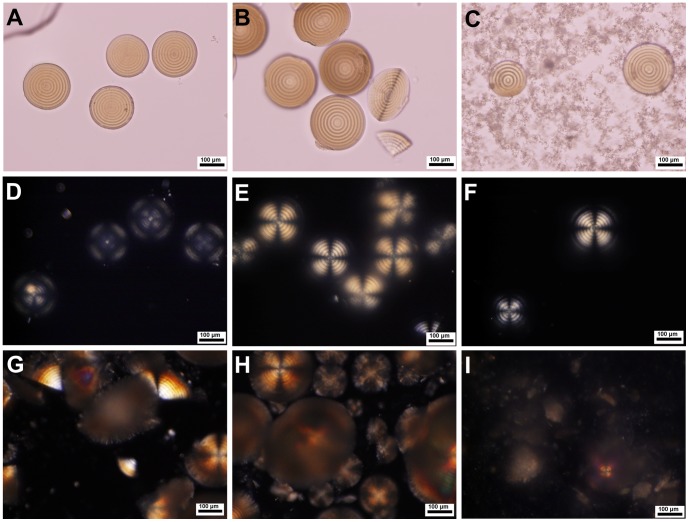
Optical microscopic images of magnesium carbonate microhemispheres synthesized from magnesium bicarbonate with different initial pH values under temperature oscillation condition. (A), (B) and (C) were transmitted light images of mineral generation from mixtures of 120 mM MgCl_2_ with 300 mM NaHCO_3_ of pH 8.4, or adjusted pH 9.0 and 9.2 (V/V, 1∶1) for 5.3 h of incubation at *T* setting of 55°C. (D), (E) and (F) were the corresponding cross-polarized light images of mineral generations same as (A), (B) and (C), respectively. (G) and (H) were cross-polaried light images of mineral generations from mineralization systems same as (A) and (B) but with incubation time of 18 h. (I) Cross-polaried light image of mineral generation from a mixture of 120 mM MgCl_2_ with 300 mM NaHCO_3_ of adjusted pH 10.7 (V/V, 1∶1) incubated at *T* setting of 5°C for 18 h.

**Figure 6:**
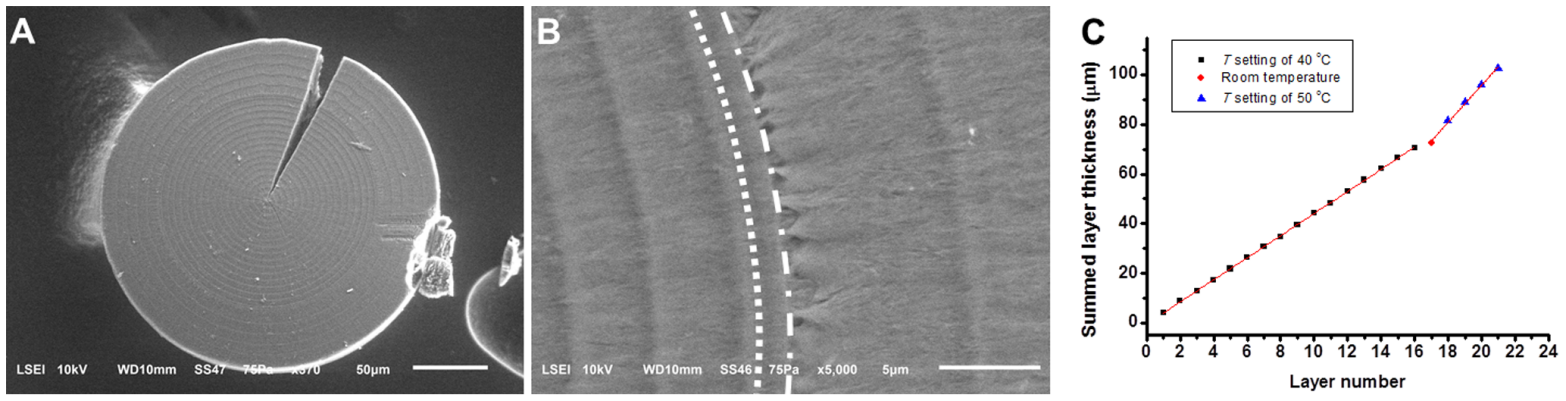
SEM images of a representative microhemisphere exposed different temperature oscillation conditions during growth. The sample was acquired from a mixture of 240_2_ and 240 mM NaHCO_3_ (1∶1, v/v) statically incubated at *T* setting of 40°C for 15 h, room temperature for 20 min, and then *T* setting of 50°C for 4 h. The last stage, corresponding to 5 temperature oscillation periods, produced the 5 thick outer layers shown in (A). (B) The magnified rings produced at the transition zone of different temperature oscillation conditions. Left side of dotted curve represents the rings formed at *T* setting of 40°C, right side of dash dotted curve represents mineral formation with temperature increased to *T* setting of 50°C. Zone between the two curves represents the mineral formation at room temperature. These three zones showed different radially aligned textures. (C) Good linear fitting of summed layer thickness measured from (A) vs. layer number curve for different temperature oscillation conditions. Slope = 4.448, R^2^ = 0.9998 for stage of *T* setting of 40°C; slope = 7.481, R^2^ = 0.9954 for stage of *T* setting of 50°C.

**Figure 7:**
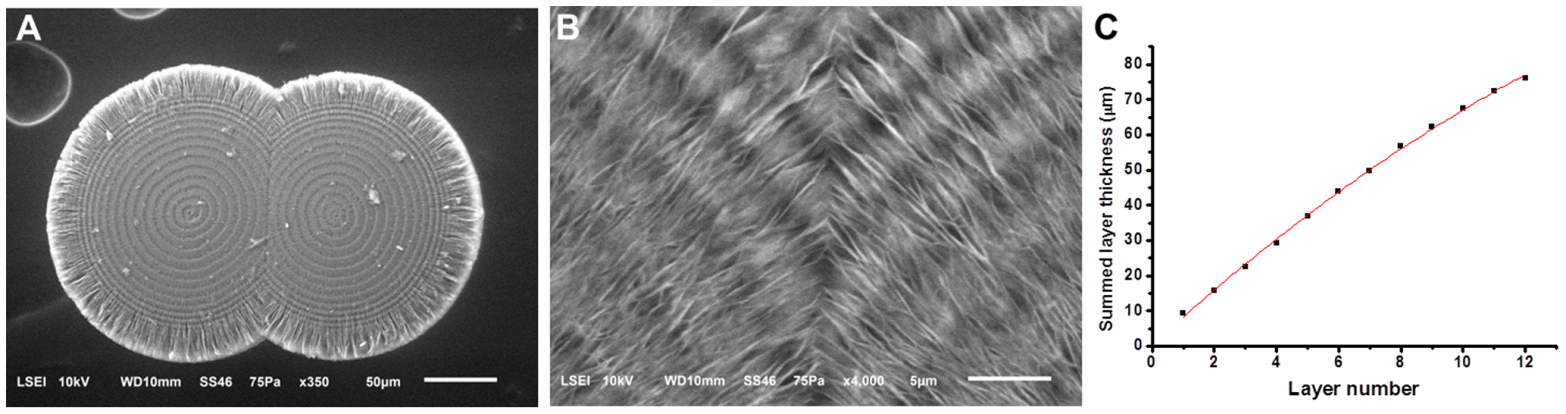
SEM images of a conjoined magnesium carbonate microparticle grown from two cores. The sample was acquired from a mxiture of 120_2_, pH 6 and 200 mM NaHCO_3_ (V/V, 1∶1) incubated at *T* setting of 50°C for 14 h. (A) Image of whole microparticle showing the two isolated cores and the intimate confluentation of outer layers. (B) Magnified confluent zone showing the synchronously formed alternating dark and light rings with similar textures. (C) Second-order polynomial fitting of summed layer thickness measured from (A) vs. layer number curves (Y = 0.3499+8.085X−0.1410X^2^, R^2^ = 0.9982).

When the magnesium bicarbonate solutions were heated at passively produced oscillatory temperatures (Fig. 2A), similar mineralization phenomena to the control experiments happened. The high concentration mineralization systems produced colloidal amorphous matter as first mineralization stage, then either rod-like microcrystals, microhemispheres or both. The systems with high initial pH values produced sticky colloidal suspension, and further generated massive tiny sphere-shaped microparticles and a small quantity of big but fragile microspheres with hard core or not. In other mineralization systems, the mineral microhemispheres appeared and slowly grew up to tens or hundreds of micrometers in diameter over time periods from about 6 hours up to 5 days at different *T* settings. However, different from what produced under thermostatic conditions, these microhemispheres synthesized under temperature oscillation conditions exhibited a distinct internal structure of alternating concentric circular dark and light rings by both optical microscopy (Figs. 2–5; Fig S3) and SEM observations (Figs. 6 and 7; Fig S4). The characteristic Maltese cross extinction patterns and concentric rings displayed by cross-polarized light microscopy were similar to that of banded polymer spherulites at some degree [49]. The enhanced crystallinity of microhemispheres with incubation time due to Ostwald ripening was also displayed (Figs. 3–5). The syntheses of such characterized microhemispheres were realized with magnesium source using MgAc_2_, MgCl_2_ or MgSO_4_, suggesting the anions of these magnesium salts have no irreplaceable role in assembly of the layered mineral structure, though acetate ions affect the concentration of free Mg^2+^ by its weak coordination ability. The primary repeating unit of the hierarchical internal structure was a single layer composed of a dark ring and the neighboring light ring shown by the microscopic images. However, the single transparent layer could also be dissociated from the crushed microhemispheres (Fig. 2B; Fig S5). It was also observed that the multi-layered structure of both wet microhemispheres kept with the mother solution and dried ones stored at room temperature showed no significant degradation in half a year of storage.

The different sizes of microhemispheres formed in the same reaction tube under conditions of a fixed temperature oscillation waveform had almost equal layer thickness. In a minority of situations, the layer thickness of a microhemisphere showed a slightly decreased trend with increasing layer number, excluding the fibrous-like shell under optical microscope if it was present, and a second-order polynomial fitting was better than linear fitting (see Figs. 6C and 7C for examples). The maximal layer number of microhemispheres was always constrained by the period number of the temperature oscillation until the mineralization reaction approached termination. Microhemispheres with tens of layers could be conveniently produced under the oscillatory temperature conditions (Fig. 2). The time to grow a single layer of the periodic microstructure was found equal to one temperature oscillation period by observation the microhemispheres growing under artificially varied temperature oscillation conditions (Fig. 6). Conjoined microhemispheres with multiple cores displayed synchronous formation of the alternating dark and light rings from different cores (Fig. 7). These results confirmed that the layered growth of magnesium carbonate microhemispheres was modulated by the temperature oscillation condition. Therefore, the distinctive multi-layered microstructure is a thermal marker of growth history of the microhemispheres.

The average growth speed of layered mineral microstructure, calculated from the temperature oscillation period and layer thickness data demonstrated in Fig. 2, was 0.5–6.7 nm/s. This mineralization speed was at a low level, implying the condition of low saturation of magnesium carbonate in solution or slow transformation of amorphous matter into crystalline microhemispheres. Both higher temperature and higher magnesium bicarbonate concentration contribute to relatively faster mineralization. But the mineralization process is in a complicated staging way. The high reactant concentration does not simply mean the faster growth of microhemispheres, as the colloidal amorphous matter can be produced in first stage as metastable intermediate and the growth of microhemispheres may encounter the competition from rod-like crystals. It's not surprising that microhemispheres of similar layer thickness were synthesized from certain systems of different concentrations under same incubation condition (Fig. 4).

The pH values of the incubated bicarbonate solutions in which magnesium carbonate microhemispheres were produced were in a range of 7.8–9.3, generally not exceeding one pH unit from that of the initial mixtures. This weak basic self-buffered reaction condition may contribute to the slow synthesis of magnesium carbonate microparticles. In the mineralization experiment with pH modified NaHCO_3_ used, the multi-layered microhemispheres were able to be produced in relatively low initial pH intervals (Fig. 5), but was hindered with increasing initial pH. However, optical microscope observation of the microspheres formed at low productivity inside the sticky colloidal suspension of these mineralization systems found that portion of them had a crystalline spherical core of multi-layered structure (Fig. S6).

**Figure 8:**
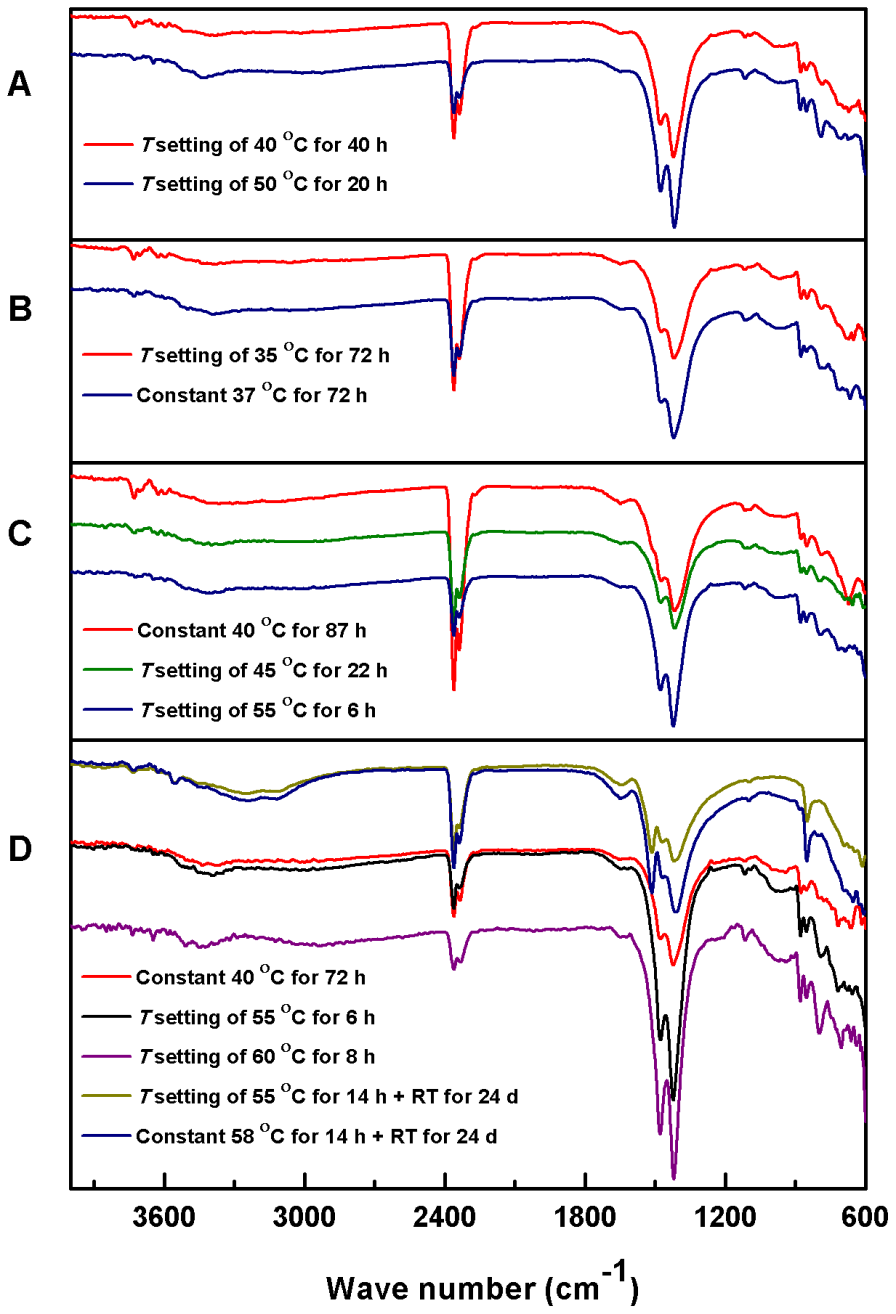
FT-IR spectra of magnesium carbonate microparticles synthesized under different incubation conditions. The reactants were (A) a mixture of 120 mM MgAc_2_, pH 6.0 and 200 mM NaHCO_3_ (1∶1, V/V), (B) a mixture of 240 mM MgCl_2_ and 450 mM NaHCO_3_ (1∶1, V/V), (C) a mixture of 240 mM MgCl_2_ and 240 mM NaHCO_3_ (1∶1, V/V) and (D) a mixture of 240 mM MgCl_2_ and 500 mM NaHCO_3_ (1∶1, V/V), respectively. The *T* setting represents the apparent instrumental temperature setting of water bath with actual oscillatory temperatures and RT represents room temperature in the legend. The IR absorption bands including three split bands around 800, 850 and 880 cm^−1^, neighboring bands around 1420 and 1480 cm^−1^, and single band around 1115 cm^−1^ are assignable to hydromagnesite, while the bands including single 850 cm^−1^ band, 1098 cm^−1^ band and three split bands around 1514, 1470 and 1410 cm^−1^ are assignable to nesquehonite.

FT-IR spectroscopy has been extensively used in the chemical structure characterization of different magnesium carbonate hydrates, which are present in hydrated or basic forms [32], [33], [50], [51]. The basic form of magnesium carbonate, hydromagnesite is more common in nature than the hydrated form, nesquehonite; the latter can readily precipitate from magnesium bicarbonate at room temperature, whereas it is metastable and converts readily to hydromagnesite [34]. Temperature plays a critical role in the magnesium carbonate mineralization by affecting the solubility of CO_2_, hydrolysis of bicarbonate ions, supersaturation of carbonate solution, crystal nucleation and growth rate, and the crystal phase [33], [35]–[37]. The nesquehonite – hydromagnesite transformation can occur at 52°C – 65°C [33], [34], [52], whereas other parameters, including CO_2_ pressure, pH value, ionic strength of solution, and crystallization kinetics also exert significant effect on this transformation [33], [37], [53]. The FT-IR spectra of the magnesium carbonate microhemispheres synthesized under different conditions were measured (Fig. 8). Most of the magnesium carbonate samples exhibited the characteristic FT-IR absorption bands assignable to hydromagnesite, including three split bands around 800, 850 and 880 cm^−1^ (CO_3_^2−^ ν_2_ bending vibrations), neighboring 1420 and 1480 cm^−1^ bands (CO_3_^2−^ ν_3_ asymmetric stretching vibrations), and 1115 cm^−1^ band (CO_3_^2−^ ν_1_ symmetric stretching vibration) (Fig. 8), indicating the layered microstructure of microhemisphere was self-assembled by basic magnesium carbonate synthesized under different conditions. The presence of H_2_O was confirmed by the shoulder band at 1645 cm^−1^ (H_2_O bending vibration). However, the quite weak IR absorption bands around 1514 cm^−1^ (CO_3_^2−^ ν_3_ asymmetric stretching vibration) and 1098 cm^−1^ (CO_3_^2−^ ν_1_ symmetric stretching vibration) assignable to nesquehonite were rarely observed in microhemispheres synthesized at low temperature, such as those synthesized at constant 40°C shown in Fig. 8C, implying the synthesis may approach the interval of co-existence of hydromagnesite with nesquehonite. The FT-IR spectra showed no significant evidence of nesquehonite – hydromagnesite transformation in samples synthesized under different temperature conditions. Moreover, if this time-related transformation was present, it should have a global effect on the mineral layers after their synthesis. However, the well preserved multi-layered structure of microhemispheres indicated that no observable degradation of the layered structure was caused by possible post-synthesis chemical transformation. On the other hand, the hydromagnesite – nesquehonite transformation was observed during the storage of multi-layered microhemispheres with the mother solution at room temperature as shown by the appearance of distinct IR absorption bands of nesquehonite, such as single 850 cm^−1^ band, 1098 cm^−1^ band and three split bands around 1514, 1470 and 1410 cm^−1^ (Fig. 8D). However, the layered mineral structure was still vivid, indicating the aging at room temperature did not significantly degrade the layered structure of synthesized magnesium carbonate microhemispheres, though chemical transformation could be involved.

**Figure 9:**
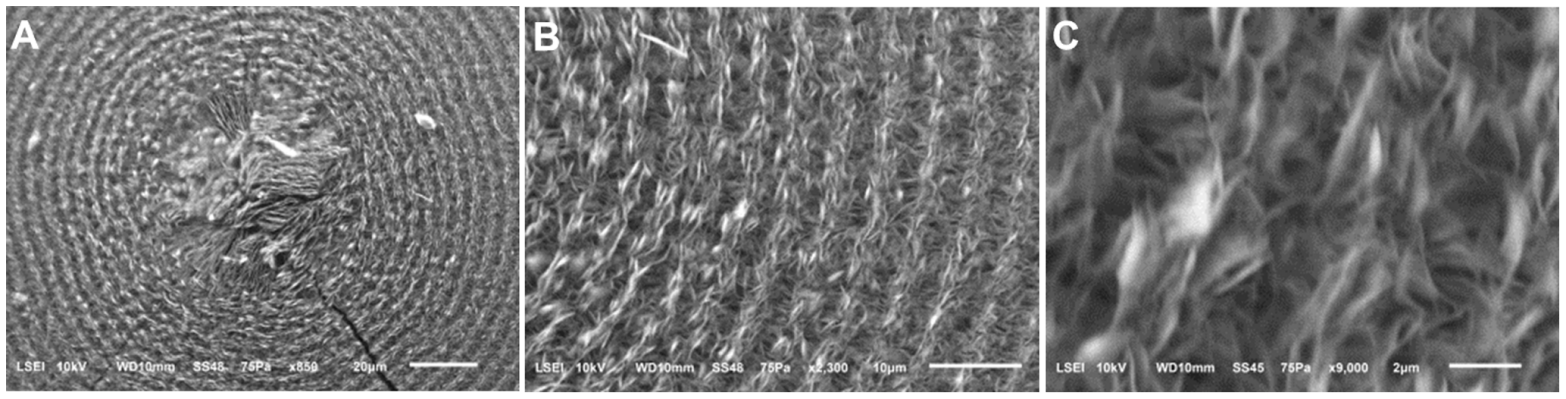
SEM images of a microhemisphere showing dark and light rings composed of radially and tangentially interlaced platelets. The reactant was a mixture of 120_2_, pH 6.0 and 200 mM NaHCO_3_ (1∶1, v/v) at *T* setting of 40°C. (A) The core composed of disorderly aligned platelets. (B) and (C) Magnified images of alternating dark and light rings composed of radially aligned short platelets and tangentially aligned long platelets, respectively, which were distorted and interlaced.

**Figure 10:**
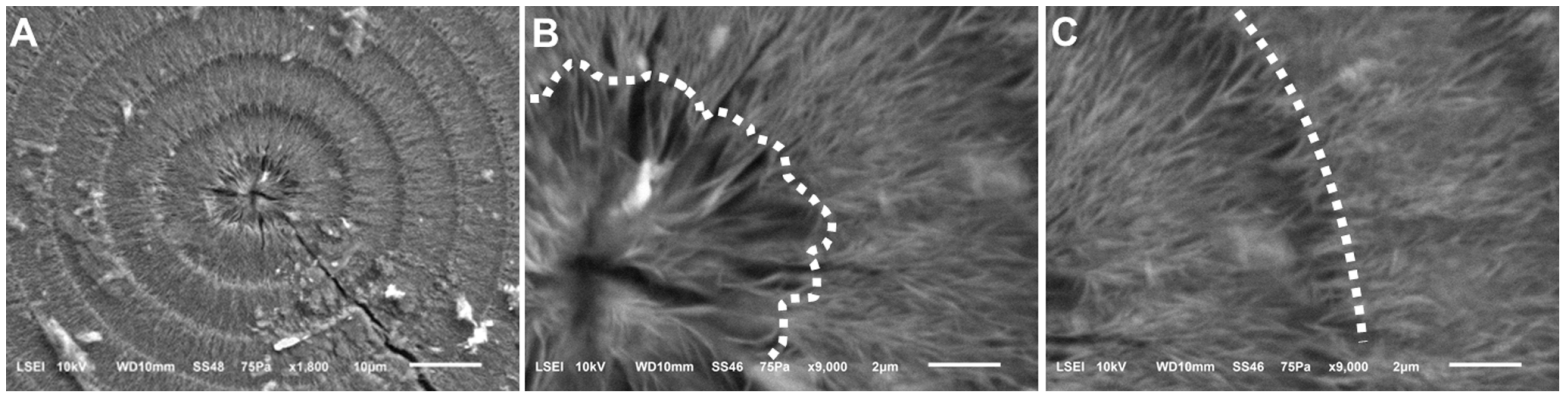
SEM images of a microhemisphere showing texture of rings composed of radially aligned platelets. The reactant was a mixture of 120_2_, pH 6.5 and 200 mM NaHCO_3_ (1∶1, v/v) at *T* setting of 50°C. (A) The microhemisphere having roughly circular core and well circular-shaped outer rings. (B) Magnified image of the core composed of roughly radially-aligned platelets with branches. (C) Magnified image of the rings showed similar texture feature as the core. Furthermore, the light rings, especially in their early growth stage (area near the right outside of dotted curves), were densely stacked by much more branched platelets, compared to the adjacent dark rings.

SEM images of the microhemispheres clearly showed the different loose mineral textures formed under different temperature oscillation conditions. The innermost core of the multi-layered microhemispheres was composed of radially aligned platelets with different degrees of distortion (Figs. 9 and 10). The dark rings of the layered structure were generally composed of radially-aligned platelets, whereas the light rings were composed of either radially-aligned or roughly tangentially- aligned platelets, affected by the temperature oscillation conditions (Figs. 9 and 10). The abrupt change of texture features leading to the formation of a boundary of mineralization rings could be observed at the artificially produced inflection point of a temperature oscillation wave (Fig. 6B). SEM images also showed the mineral layers of these same microhemispheres generally displayed similar characteristic textures, in spite of their distance from the core (Fig. 9), indicating their architectural stability post synthesis. Obviously, the formation of these periodically-layered magnesium carbonate microhemispheres with complex texture was very likely not dominated by the classic ion-by-ion attachment crystallization pathway.

**Figure 11:**
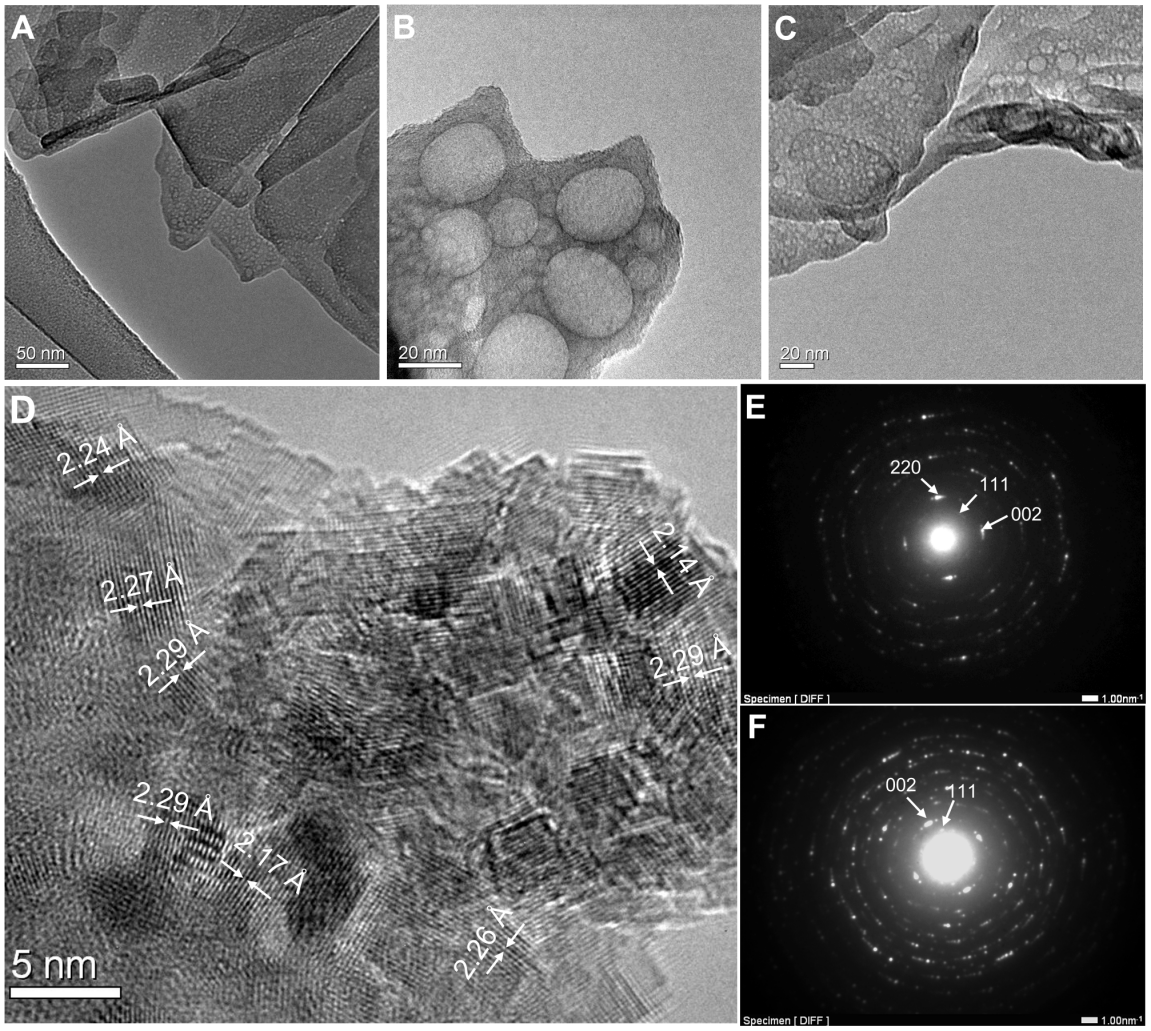
TEM images and ED patterns of crushed fragments of synthesized magnesium carbonate microhemispheres. (A) and (B) TEM images of sample synthesized from a mixture of 120 mM MgAc_2_, pH 6.0 with 200 mM NaHCO_3_ (V/V, 1∶1) at *T* setting of 55°C for 7 h. The corresponding ED pattern was shown in (E). (C) and (D) TEM and HRTEM images of sample synthesized from a mixture of 120 mM MgCl_2_ with 300 mM NaHCO_3_ of adjusted pH 9.0 (V/V, 1∶1) at *T* setting of of 55°C for 18 h. The corresponding ED pattern was shown in (F). Hydromagnesite of polycrystalline structure with some degree of oritentation was affirmed from the ED dots and arcs and characteristic interplanar spacings derivated from them.

TEM observation of the crushed and ultrasonicated fragments of microhemispheres found the mineral texture was assembled by flakes (Fig. 11). A large number of mesopores with variable diameters (roughly < 10 nm) were randomly present within the flakes. The mesopores of microhemispheres synthesized in short time of incubation, which usually had low crystallinity, were enlarged and the structure were further damaged by electron beam irradiation during high resolution TEM (HRTEM) imaging, however, the microhemispheres with high crystallinity had relatively stable mesopores under HRTEM and a structure of aggregated assembly of nanoflakes (width of several nanometers) (Fig. 11D). A lattice fringe spacing of 2.3 Å was acquired from the HRTEM images, probably corresponding to that of the (800) plane of hydromagnesite (Fig. 11D). The ED patterns affirmed the nanoflakes of microhemispheres were of polycrystalline structure with some degree of oriented assembly (Fig. 11E and 11F). Hydromagnesite could be identified according to the characteristic lattice spacing calculated from the ED spots and arcs (Fig. 11E and 11F).

**Figure 12:**
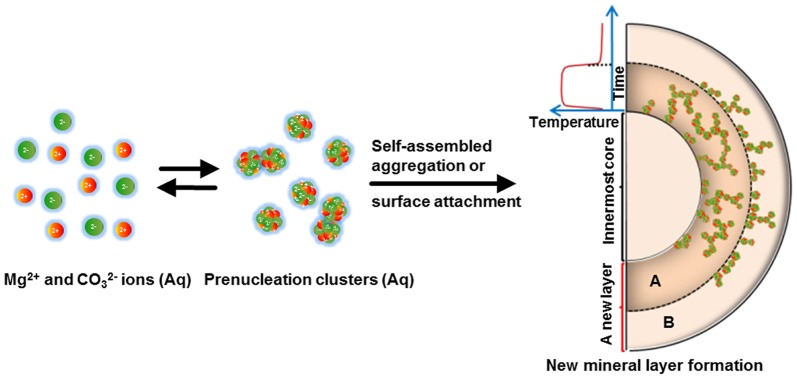
Schematic of the layered growth mechanism of magnesium carbonate microhemispheres. Pre-nucleation nanoclusters of magnesium carbonate are produced in the slightly supersaturated solution under heating condition. These nanoclusters can directly attach on preformed mineral surface, or expose self-assembled aggregation to form new nuclei and mineral intermediates with morphology preforming property. The varying temperature disturbs the equilibrium of these two pathways adjacent to mineralization front, and finally leads to different heterogeneous mineral textures. Higher temperature accelerates the reaction and favors production of more nanoclusters, and thus increases the odds of aggregated nucleation pathway (ring A), on the contrary, lower temperature favors production of smaller amount of nanoclusters and surface attachment pathway (ring B). A significant mineralization boundary may appear around the temperature inflection point. A single layer, composed of two neighboring rings with different texture characteristics, can be formed corresponding to a temperature oscillation period.

We speculate the pre-nucleation nanoclusters of magnesium carbonate [47], [48] produced in the supersaturated solutions of incubated magnesium bicarbonate play an important role in the multi-layered mineralization. These nanoclusters can follow two low-energy pathways: 1) direct attachment on the surface of a preformed mineral core, and 2) self-assembled aggregation to nucleation adjacent to the mineral surface (schematic in Fig. 12). The oriented attachment of neighboring nuclei and further growth can build up distinct, oriented amorphous intermediates before transition to stable polymorphs [3], [17], [20]–[22]. Thus, the mineralization texture is controlled by the synergistic action of these two competitive pathways, while the classic ion-attachment pathway may also be involved at some degree, depending on the specific temperature conditions. The equilibrium of this synergistic action is maintained under thermostatic conditions, but is disturbed by non-equilibrium thermodynamics. As temperature determines the mineralization speed, more nanoclusters are assumed to be produced at higher temperature and thus increase the odds of the aggregated nucleation pathway (ring A in Fig. 12); on the contrary, the decreased mineral saturation at lower temperature may favor the surface attachment pathway rather than the clustering aggregation pathway (ring B in Fig. 12). The different characteristic mineral textures are formed by the non-equilibrated coordination of these pathways at different temperatures, consequently leading to the periodic mineral layers under temperature oscillation conditions. Though detailed chemical compositions of the nanoclusters and mineral intermediates are not described in this study, their presence in magnesium bicarbonate solutions has been demonstrated in literatures [47], [48] and implied by the characteristic internal texture and oriented nano-polycrystalline structure of microhemispheres exhibited by TEM images and ED patterns. The FT-IR data support the hypothesis that mainly the basic form of magnesium carbonate participated in the layered mineralization at different *T* settings.

**Figure 13:**
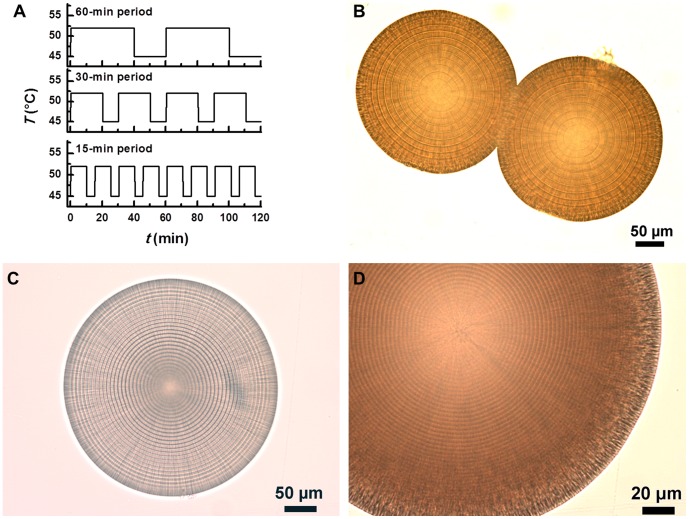
Magnesium carbonate microhemispheres synthesized with a programmable PCR thermocycler. The reactant was a mixture of 240_2_ and 500 mM NaHCO_3_ (1∶1, V/V). (A) Similar temperature oscillation patterns having different periods. (B–D) Transmitted light microscopic images of microhemispheres produced by the temperature oscillation patterns with 60, 30 and 15-min period, respectively. The corresponding layer thicknesses were 12.0±0.2, 6.0±0.1 and 2.6±0.0 µm.

Evidently, both high temperature and a long period temperature oscillation waveform are factors favoring the growth of a thick mineral layer of microhemisphere. The experiments under passively produced temperature oscillation conditions indicate that a higher temperature setting combined with a shorter oscillation period could lead to a similar layer thickness as a lower temperature setting combined with a longer oscillation period (Fig. 2D vs. 2E, Fig. 2F vs. 2G). This speculation was further tested by the observation of multi-layered microhemispheres synthesized with a PCR thermocycler using different incubation settings. As expected, the layer thickness decreased from 12.0 to 2.6 µm when the oscillation period was decreased from 60 minutes to 15 minutes, at a constant average temperature and a constant oscillation amplitude (a simple square wave shape was arbitrarily chosen for this experimental trial) (Fig. 13).

Our experiments verified the modulation effect of temperature oscillation on the self-assembly of mineral microstructure in solution. Appropriate reactant concentration and initial pH can restrain the competitive growth of other mineral generations. The relatively slow mineralization reaction and static growth allow the mineralization of magnesium carbonate to occur as a multi-layered self-assembly synchronized to the oscillatory temperatures. Further studies are required to explore the detailed mechanism of such thermodynamic modulation, which will contribute to our comprehensive understanding of the non-equilibrium mineralization process. The work described in this paper does show, however, that temperature oscillation modulation, as a non-invasive and programmable method, has the potential to combine with other synthetic techniques using chemical templates, additives or gel-diffusion systems [3], [17], [26] for the preparation of hierarchically ordered mineral materials.

## Conclusions

In conclusion, temperature oscillation was verified to be a technique able to precisely modulate the self-assembly of rhythmic concentric layered magnesium carbonate microparticles. This novel temperature oscillation modulated self-assembly technique may give new insight to understanding the genesis of natural, concentric-layered mineral microparticles. The non-invasive and programmable properties of temperature oscillation also reveal it as a potential technique to combine with other synthetic approaches using chemical mediators for the preparation of hierarchically ordered mineral materials.

## Supporting Information

Figure S1 **Optical microscopic images of mineral synthesized in 333 mM magnesium bicarbonate under thermostatic condition of 55°C.** The magnesium bicarbonate was prepared by mixing of MgCl_2_ and NaHCO_3_ (molar ratio, 1∶2). (A) Clustered rod-like crystals produced from the metastable amorphous suspension in a reactant tube after 5 h of incubation. (B) Cross-polarized light image of sample from (A) affirmed the amorphous phase (dark view) and the crystal phase (light view). (C) and (D) Transmitted light image and cross-polarized light image of microhemispheres produced by 44 h of incubation and crushed fragments. (E) and (F) Transmitted light image and cross-polarized light image of microhemispheres produced by 5 d of incubation and crushed fragments.
(TIF)

Figure S2 **Optical microscopic images of mineral synthesized in magnesium bicarbonate of high pH values under thermostatic condition of 55°C.** (A) Mineral generation of mixtures of 120 mM MgAc_2_ with 300 mM NaHCO_3_ (V/V, 1∶1) after 18 h of incubation. The pH values of NaHCO_3_ component were 9.0, 9.2, 10.0 and 10.7, respectively, for the reaction tubes from left to right marked as Y2, Y3, Y4 and Y5. Colloidal suspension was present in mineralization systems with higher initial pH values (Y4 and Y5). (B) and (C) Transmitted light and cross-polarized light images of amorphous matter from mineralization system (Y4) after 1.5 h of incubation. (D) and (E) Cross-polarized light images of colloidal sample from mineralization system (Y4) after 18 h and 2.5 d of incubation. The appearance of many crystalline cores from amorphous phase and formation of fragile microspheres of bigger sizes with incubation time were displayed. Characteristic Maltese cross extinction appeared with the crystalline core of big microspheres. (F) and (G) Transmitted light image and cross polarized light image of mineralization system (Y5) after 18 h of incubation. The massive sphere-like microparticles transformed from amorphous matter displayed significant crystalline cores. (H) Cross-polarized light image of (Y5) after 2.5 d of incubation displayed the presence of fragile crystalline microspheres of big sizes together with agglomerates of massive small microparticles. (I) and (J) Transmitted light image and cross-polarized light image of mineral generation from a mixture of 160 mM MgSO_4_ with 333 mM NaHCO_3_, pH 10.7 (V/V, 1∶1) after 2 h of incubation. A large number of tiny crystalline microspheres were transformed from the amorphous matter.
(TIF)

Figure S3 **Reflected light microscopic images of multi-layered magnesium carbonate microparticles.** (A) The dried microparticles showing the hemispherical shape. (B) The wet microparticles showing the translucent layered internal structure.
(TIF)

Figure S4 **SEM image of the major fragment of a multi-layered magnesium carbonate microhemisphere exhibiting the concentric multi-layered structure.**
(TIF)

Figure S5 **Transmitted light microscopic image exhibiting the dissociated layers of a magnesium carbonate microhemisphere.** The microhemisphere was synthesized from a mixture of 120 mM MgAc_2_, pH 6.5 with 240 mM NaHCO_3_ (V/V, 1∶1) incubated at *T* setting of 55°C for 6 h. (A) Intact microhemisphere. (B) Crushed fragments.
(TIF)

Figure S6 **Microspheres synthesized in colloidal suspension of a mineralization system with high initial pH under temperature oscillation condition.** The product was from a mixture of 240 mM MgSO_4_ with 500 mM NaHCO_3_ of adjusted pH 10.7 (V/V, 1∶1) incubated at *T* setting of 55°C for 18 h. (A) Transmitted light image showing the structure of fibrous shell and transparent multi-layered core. (B) Cross-polarized light image showing Maltese cross extinction of crystalline and layered core with shell stripped.
(TIF)

## References

[1] Ball P (1999) The self-made tapestry: pattern formation in nature. Oxford England; New York: Oxford University Press. vi, 287 p.

[2] Whitesides GM, Grzybowski B (2002) Self-assembly at all scales. Science 295: 2418–2421. 11923529 10.1126/science.1070821

[3] Mann S (2009) Self-assembly and transformation of hybrid nano-objects and nanostructures under equilibrium and non-equilibrium conditions. Nature Materials 8: 781–792. 19734883 10.1038/nmat2496

[4] Bathurst RGC (1979) Carbonate sediments and their diagenesis. Amsterdam, New York: Elsevier. xix, 658 p.

[5] Davies PJ, Bubela B, Ferguson F (1978) The formation of ooids. Sedimentology 25: , 703–730.

[6] Folk RL, Lynch FL (2001) Organic matter, putative nannobacteria and the formation of ooids and hardgrounds. Sedimentology 48: 215–229.

[7] Duguid SMA, Kyser TK, James NP, Rankey EC (2010) Microbes and ooids. Journal of Sedimentary Research 80: 236–251.

[8] Pacton M, Ariztegui D, Wacey D, Kilburn MR, Rollion-Bard C, et al (2012) Going nano: A new step toward understanding the processes governing freshwater ooid formation. Geology 40: 547–550.

[9] Kleiner O, Ramesh J, Huleihel M, Cohen B, Kantarovich K, et al (2002) A comparative study of gallstones from children and adults using FTIR spectroscopy and fluorescence microscopy. BMC gastroenterology 2: 3. 11872150 10.1186/1471-230X-2-3 PMC65695

[10] Campana SE (1984) Microstructural growth-patterns in the otoliths of larval and juvenile starry flounder, Platichthys stellatus. Canadian Journal of Zoology-Revue Canadienne De Zoologie 62: 1507–1512.

[11] Volk EC, Schroder SL, Grimm JJ (1999) Otolith thermal marking. Fisheries Research 43: 205–219.

[12] Tuur SM, Nelson AM, Gibson DW, Neafie RC, Johnson FB, et al (1987) Liesegang rings in tissue - How to distinguish Liesegang rings from the giant kidney worm, Dioctophyma renale. American Journal of Surgical Pathology 11: 598–605. 2956899

[13] Tong H, Wan P, Ma WT, Zhong GR, Cao LX, et al (2008) Yolk spherocrystal: The structure, composition and liquid crystal template. Journal of Structural Biology 163: 1–9. 18485735 10.1016/j.jsb.2008.03.012

[14] Henisch HK (1988) Crystals in gels and Liesegang rings : in vitro veritas. Cambridge England; New York: Cambridge University Press. xiii, 197 p. 10.1126/science.242.4885.1585-a 17788426

[15] Grzybowski BA, Bishop KJM, Campbell CJ, Fialkowski M, Smoukov SK (2005) Micro- and nanotechnology via reaction-diffusion. Soft Matter 1: 114–128.

[16] Banfield JF, Welch SA, Zhang HZ, Ebert TT, Penn RL (2000) Aggregation-based crystal growth and microstructure development in natural iron oxyhydroxide biomineralization products. Science 289: 751–754. 10926531 10.1126/science.289.5480.751

[17] Colfen H, Mann S (2003) Higher-order organization by mesoscale self-assembly and transformation of hybrid nanostructures. Angewandte Chemie-International Edition 42: 2350–2365. 12783497 10.1002/anie.200200562

[18] Gebauer D, Volkel A, Colfen H (2008) Stable prenucleation calcium carbonate Clusters. Science 322: 1819–1822. 19095936 10.1126/science.1164271

[19] Pouget EM, Bomans PHH, Goos JACM, Frederik PM, de With G, et al (2009) The initial stages of template-controlled CaCO_3_ formation revealed by cryo-TEM. Science 323: 1455–1458. 19286549 10.1126/science.1169434

[20] Zhang Q, Liu SJ, Yu SH (2009) Recent advances in oriented attachment growth and synthesis of functional materials: concept, evidence, mechanism, and future. Journal of Materials Chemistry 19: 191–207.

[21] Yuwono VM, Burrows ND, Soltis JA, Penn RL (2010) Oriented aggregation: Formation and transformation of mesocrystal intermediates revealed. Journal of the American Chemical Society 132: 2163–2165. 20112897 10.1021/ja909769a

[22] Gebauer D, Colfen H (2011) Prenucleation clusters and non-classical nucleation. Nano Today 6: 564–584.

[23] Demichelis R, Raiteri P, Gale JD, Quigley D, Gebauer D (2011) Stable prenucleation mineral clusters are liquid-like ionic polymers. Nature Communications 2. Available: http://www.nature.com/ncomms/journal/v2/n12/pdf/ncomms1604.pdf. Accessed 5 September 2013. 10.1038/ncomms1604 PMC3247826 22186886

[24] Sear RP (2012) The non-classical nucleation of crystals: microscopic mechanisms and applications to molecular crystals, ice and calcium carbonate. International Materials Reviews 57(6): 328–356.

[25] Gilbert B, Lu G, Kim CS (2007) Stable cluster formation in aqueous suspensions of iron oxyhydroxide nanoparticles. Journal of Colloid and Interface Science 313: 152–159. 17511999 10.1016/j.jcis.2007.04.038

[26] Meldrum FC, Colfen H (2008) Controlling mineral morphologies and structures in biological and synthetic systems. Chemical Reviews 108: 4332–4432. 19006397 10.1021/cr8002856

[27] Chen SF, Yu SH, Jiang J, Li FQ, Liu YK (2006) Polymorph discrimination of CaCO_3_ mineral in an ethanol/water solution: Formation of complex vaterite superstructures and aragonite rods. Chemistry of Materials 18: 115–122.

[28] Jiang J, Chen SF, Liu L, Yao HB, Qiu YH, et al. (2009) Template-free polymorph discrimination and synthesis of calcium carbonate minerals. Chemical Communications: 5853–5855. 10.1039/b911219g 19787119

[29] Jiang JX, Ye JZ, Zhang GW, Gong XH, Nie LH, et al (2012) Polymorph and Morphology Control of CaCO_3_ via Temperature and PEG During the Decomposition of Ca(HCO_3_)_2_ . Journal of the American Ceramic Society 95: 3735–3738.

[30] Liu YY, Jiang J, Gao MR, Yu B, Mao LB, et al (2013) Phase Transformation of Magnesium Amorphous Calcium Carbonate (Mg-ACC) in a Binary Solution of Ethanol and Water. Crystal Growth & Design 13: 59–65.

[31] Du J, Chen Z, Wu YL, Yang MD, Dang J, et al (2013) Study on crystal transformation process of magnesium carbonate hydrate based on salt lake magnesium resource utilization. Turkish Journal of Chemistry 37: 228–238.

[32] Botha A, Strydom CA (2003) DTA and FT-IR analysis of the rehydration of basic magnesium carbonate. Journal of Thermal Analysis and Calorimetry 71: 987–996.

[33] Zhang ZP, Zheng YJ, Ni YW, Liu ZM, Chen JP, et al (2006) Temperature- and pH-dependent morphology and FT-IR analysis of magnesium carbonate hydrates. Journal of Physical Chemistry B 110: 12969–12973. 10.1021/jp061261j 16805601

[34] Hopkinson L, Rutt K, Cressey G (2008) The transformation of nesquehonite to hydromagnesite in the system CaO-MgO-H_2_O-CO_2_: An experimental FT-Raman spectroscopic study. Journal of Geology 116: 387–400.

[35] Marion GM (2001) Carbonate mineral solubility at low temperatures in the Na-K-Mg-Ca-H-Cl-SO_4_-OH-HCO_3_-CO_3_-CO_2_-H_2_O system. Geochimica Et Cosmochimica Acta 65: 1883–1896.

[36] Dong M, Cheng WT, Li ZB, Demopoulos GP (2008) Solubility and stability of nesquehonite (MgCO_3_⋅3H_2_O) in NaCl, KCl, MgCl_2_, and NH_4_Cl Solutions. Journal of Chemical and Engineering Data 53: 2586–2593.

[37] Hanchen M, Prigiobbe V, Baciocchi R, Mazzotti M (2008) Precipitation in the Mg-carbonate system - effects of temperature and CO_2_ pressure. Chemical Engineering Science 63: 1012–1028.

[38] Radha AV, Forbes TZ, Killian CE, Gilbert PUPA, Navrotsky A (2010) Transformation and crystallization energetics of synthetic and biogenic amorphous calcium carbonate. Proceedings of the National Academy of Sciences of the United States of America 107: 16438–16443. 20810918 10.1073/pnas.1009959107 PMC2944757

[39] Radha AV, Fernandez-Martinez A, Hu YD, Jun YS, Waychunas GA, et al (2012) Energetic and structural studies of amorphous Ca_1-x_Mg_x_CO_3_⋅nH_2_O (0< = x< = 1). Geochimica Et Cosmochimica Acta 90: 83–95.

[40] Politi Y, Batchelor DR, Zaslansky P, Chmelka BF, Weaver JC, et al (2010) Role of magnesium ion in the stabilization of biogenic amorphous calcium carbonate: A structure-function investigation. Chemistry of Materials 22: 161–166.

[41] Russell MJ, Ingham JK, Zedef V, Maktav D, Sunar F, et al (1999) Search for signs of ancient life on Mars: expectations from hydromagnesite microbialites, Solda Lake, Turkey. Journal Geological Society London 156: 869–888.

[42] Niles PB, Catling DC, Berger G, Chassefière E, Ehlmann BL, et al (2013) Geochemistry of carbonates on Mars: Implications for climate history and nature of aqueous environments. Space Science Reviews 174: 301–328.

[43] Oelkers EH, Schott J (2005) Geochemical aspects of CO_2_ sequestration. Chemical Geology 217: 183–186.

[44] Sipala J, Teir S, Zevenhoven R (2008) Carbon dioxide sequestration by mineral carbonation - literature review update 2005–2007. Åbo Akademi University Faculty of Technology Heat Engineering Laboratory, Report VT 2008-1. Åbo Akademis Tryckeri, Finland. pp. 1–59.

[45] Botha A, Strydom CA (2001) Preparation of a magnesium hydroxyl carbonate from magnesium hydroxide. Hydrometallurgy. 2001 62: 175–183.

[46] Truitt B (2009) Magnesium Carbonate. In Handbook of Pharmaceutical Excipients, 6th ed.; Rowe R, Sheskey P, Quinn M, Eds.; Pharmaceutical Press: London; 397–400.

[47] Verch A, Antonietti M, Colfen H (2012) Mixed calcium-magnesium pre-nucleation clusters enrich calcium. Zeitschrift Fur Kristallographie 227: 718–722.

[48] Zhao L, Zhu C, Ji JF, Chen J, Teng HH (2013) Thermodynamic and kinetic effect of organic solvent on the nucleation of nesquehonite. Geochimica Et Cosmochimica Acta 106: 192–202.

[49] Keith HD, Padden FJ (1996) Banding in polyethylene and other spherulites. Macromolecules 29: 7776–7786.

[50] White WB (1971) Infrared characterization of water and hydroxyl ion in the basic magnesium carbonate minerals. Am Mineral 56: 46–53.

[51] Sawada Y, Uematsu K, Mizutani N, Kato M (1978) Thermal decomposition of hydromagnesite 4MgCO_3_⋅Mg(OH)_2_⋅4H_2_O under different partial pressures of carbon dioxide. Thermochim Acta 27: 45–59.

[52] Davies PJ, Bubela B (1973) The transformation of nesquehonite into hydromagnesite. Chemical Geology 12: 289–300.

[53] Xiong Y, Lord AS (2008) Experimental investigations of the reaction path in the MgO–CO_2_–H_2_O system in solutions with various ionic strengths, and their applications to nuclear waste isolation. Applied Geochemistry 23: 1634–1659.

