# Supplementary figures and images for: Temperature Oscillation Modulated Self-Assembly of Periodic Concentric Layered Magnesium Carbonate Microparticles
Source: PLoS One. 2014 Feb 10;9(2):e88648. doi: 10.1371/journal.pone.0088648 (PMC3919819; doi:10.1371/journal.pone.0088648)

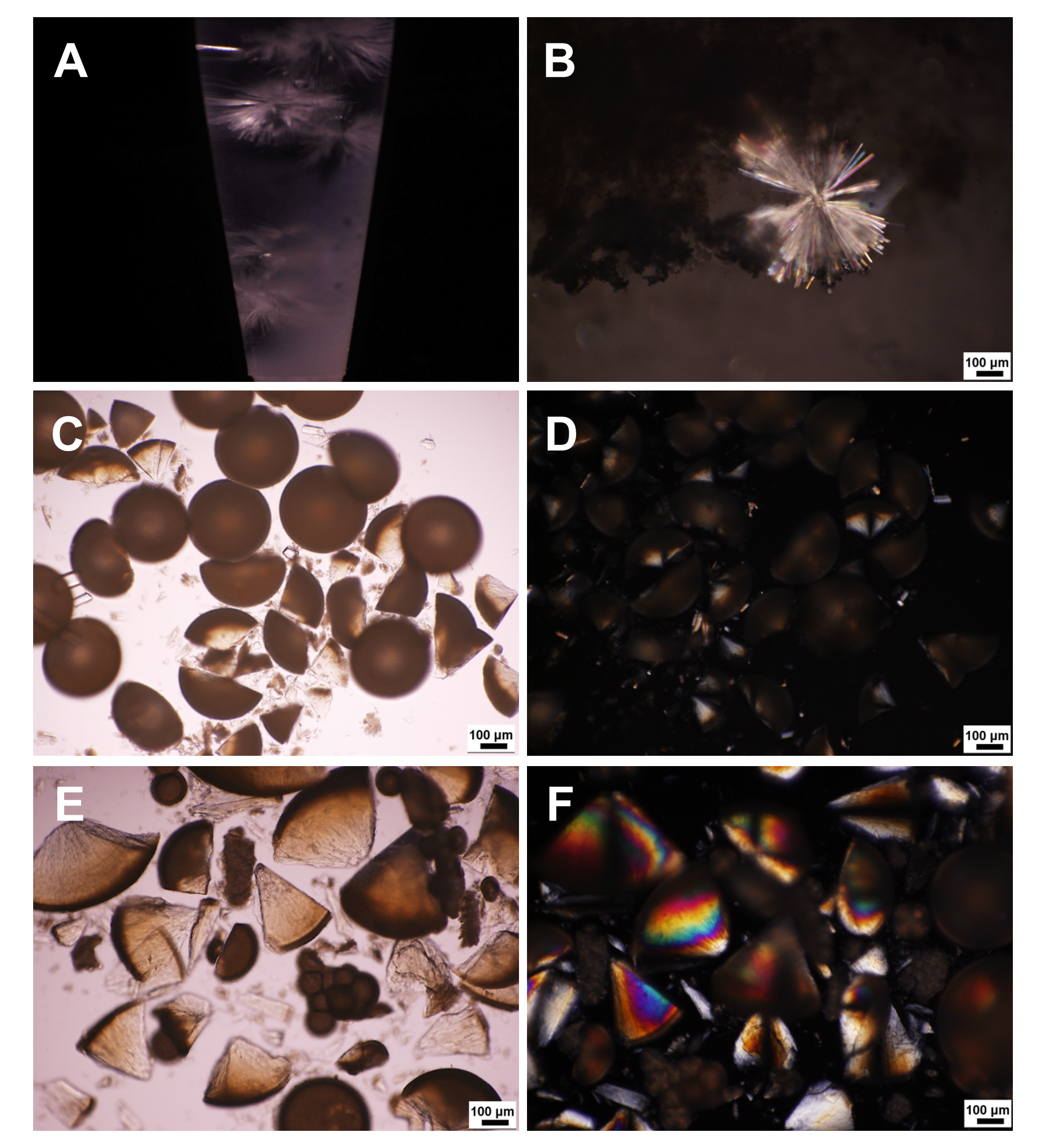

Supplement: Figure S1 — Optical microscopic images of mineral synthesized in 333 mM magnesium bicarbonate under thermostatic condition of 55°C. The magnesium bicarbonate was prepared by mixing of MgCl2 and NaHCO3 (molar ratio, 1∶2). (A) Clustered rod-like crystals produced from the metastable amorphous suspension in a reactant tube after 5 h of incubation. (B) Cross-polarized light image of sample from (A) affirmed the amorphous phase (dark view) and the crystal phase (light view). (C) and (D) Transmitted light image and cross-polarized light image of microhemispheres produced by 44 h of incubation and crushed fragments. (E) and (F) Transmitted light image and cross-polarized light image of microhemispheres produced by 5 d of incubation and crushed fragments. (TIF) [file pone.0088648.s001.tif]

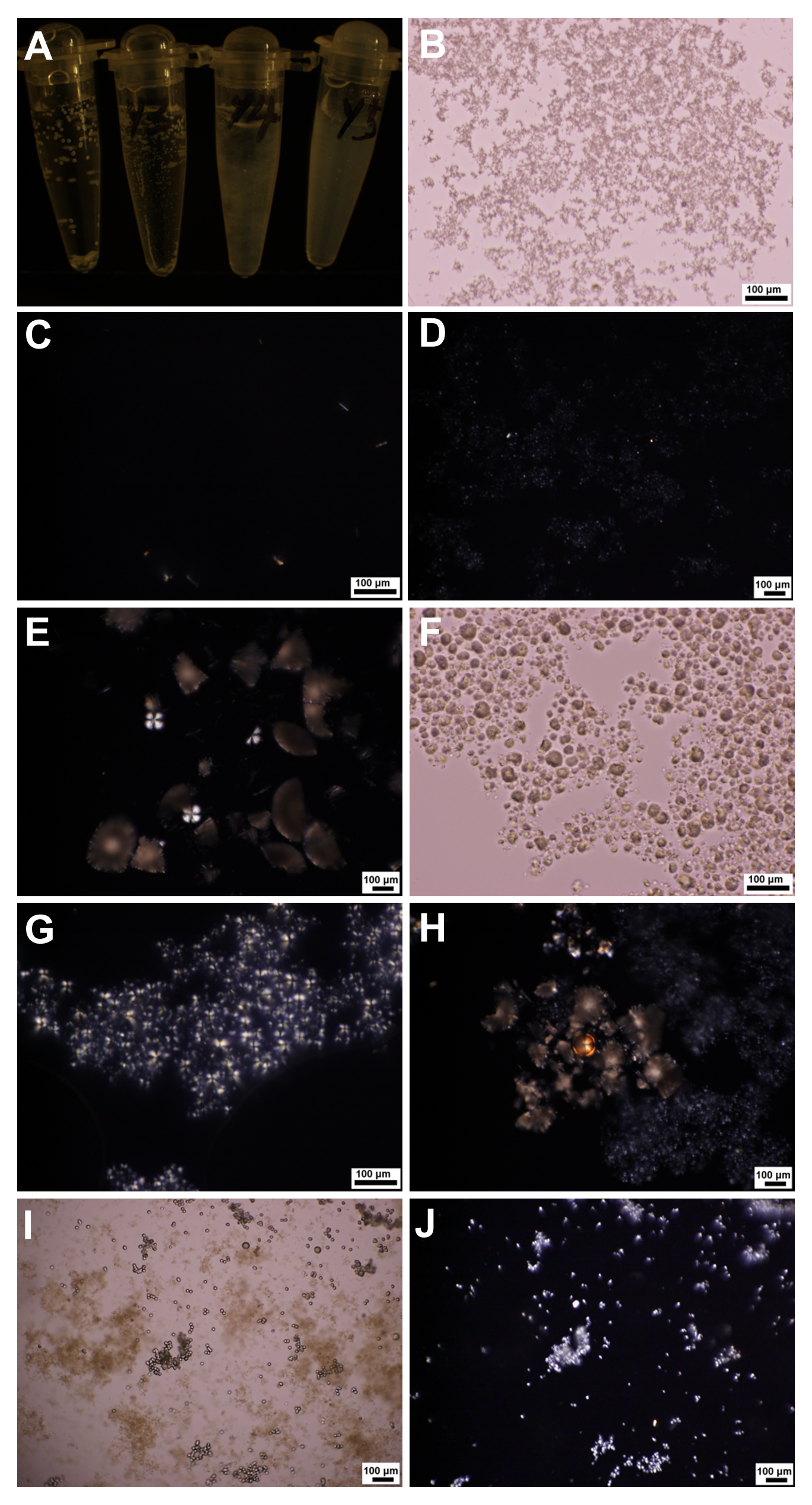

Supplement: Figure S2 — Optical microscopic images of mineral synthesized in magnesium bicarbonate of high pH values under thermostatic condition of 55°C. (A) Mineral generation of mixtures of 120 mM MgAc2 with 300 mM NaHCO3 (V/V, 1∶1) after 18 h of incubation. The pH values of NaHCO3 component were 9.0, 9.2, 10.0 and 10.7, respectively, for the reaction tubes from left to right marked as Y2, Y3, Y4 and Y5. Colloidal suspension was present in mineralization systems with higher initial pH values (Y4 and Y5). (B) and (C) Transmitted light and cross-polarized light images of amorphous matter from mineralization system (Y4) after 1.5 h of incubation. (D) and (E) Cross-polarized light images of colloidal sample from mineralization system (Y4) after 18 h and 2.5 d of incubation. The appearance of many crystalline cores from amorphous phase and formation of fragile microspheres of bigger sizes with incubation time were displayed. Characteristic Maltese cross extinction appeared with the crystalline core of big microspheres. (F) and (G) Transmitted light image and cross polarized light image of mineralization system (Y5) after 18 h of incubation. The massive sphere-like microparticles transformed from amorphous matter displayed significant crystalline cores. (H) Cross-polarized light image of (Y5) after 2.5 d of incubation displayed the presence of fragile crystalline microspheres of big sizes together with agglomerates of massive small microparticles. (I) and (J) Transmitted light image and cross-polarized light image of mineral generation from a mixture of 160 mM MgSO4 with 333 mM NaHCO3, pH 10.7 (V/V, 1∶1) after 2 h of incubation. A large number of tiny crystalline microspheres were transformed from the amorphous matter. (TIF) [file pone.0088648.s002.tif]

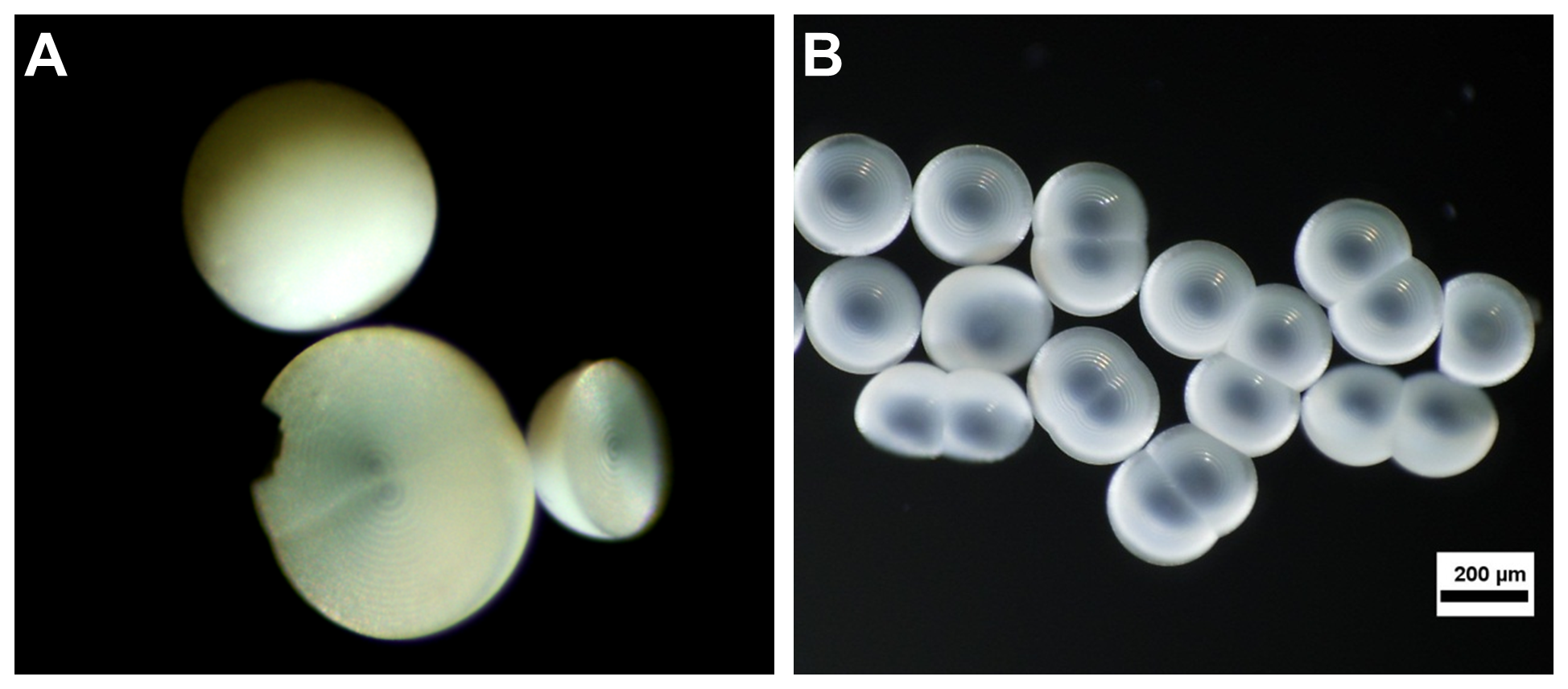

Supplement: Figure S3 — Reflected light microscopic images of multi-layered magnesium carbonate microparticles. (A) The dried microparticles showing the hemispherical shape. (B) The wet microparticles showing the translucent layered internal structure. (TIF) [file pone.0088648.s003.tif]

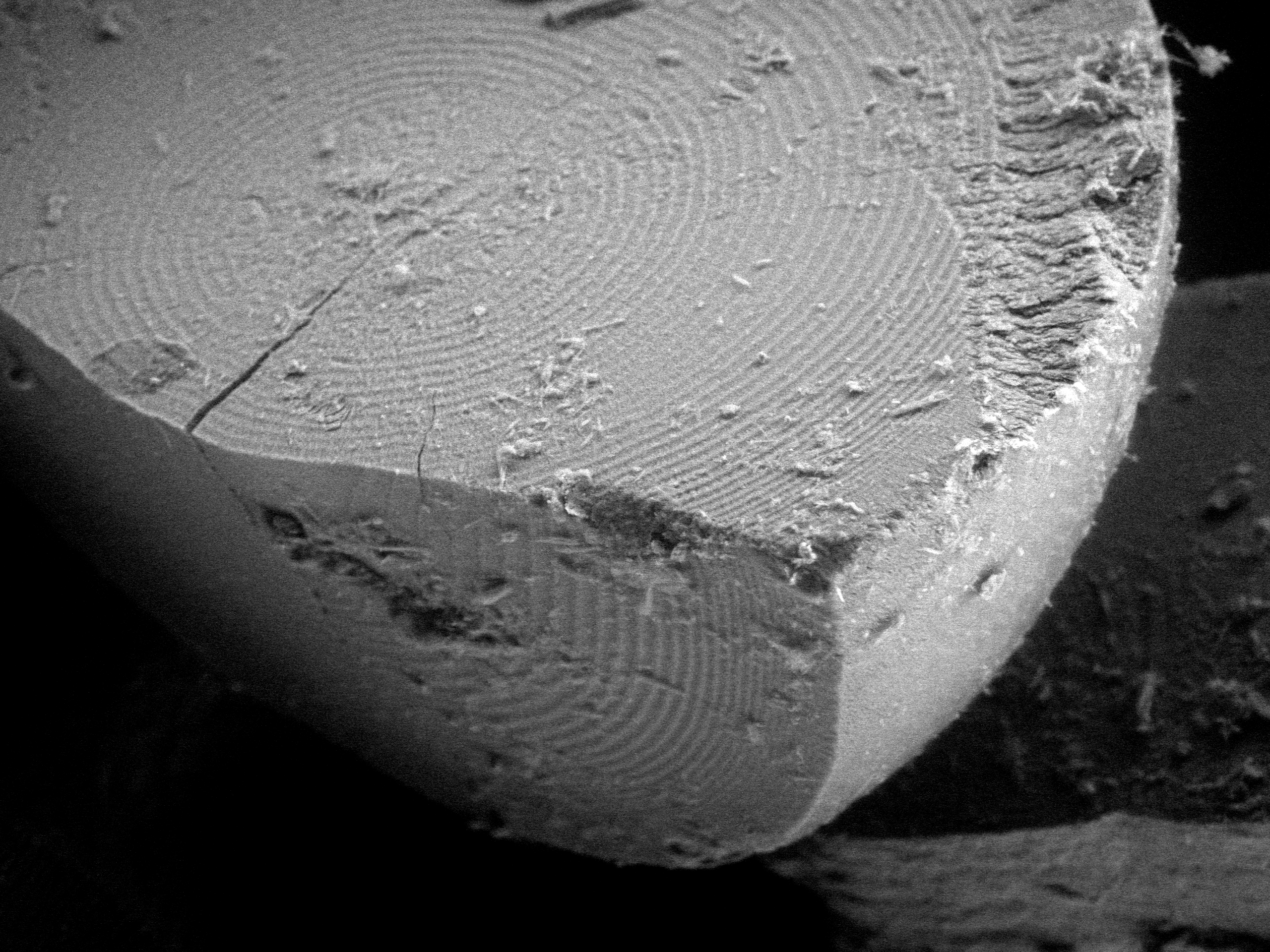

Supplement: Figure S4 — SEM image of the major fragment of a multi-layered magnesium carbonate microhemisphere exhibiting the concentric multi-layered structure. (TIF) [file pone.0088648.s004.tif]

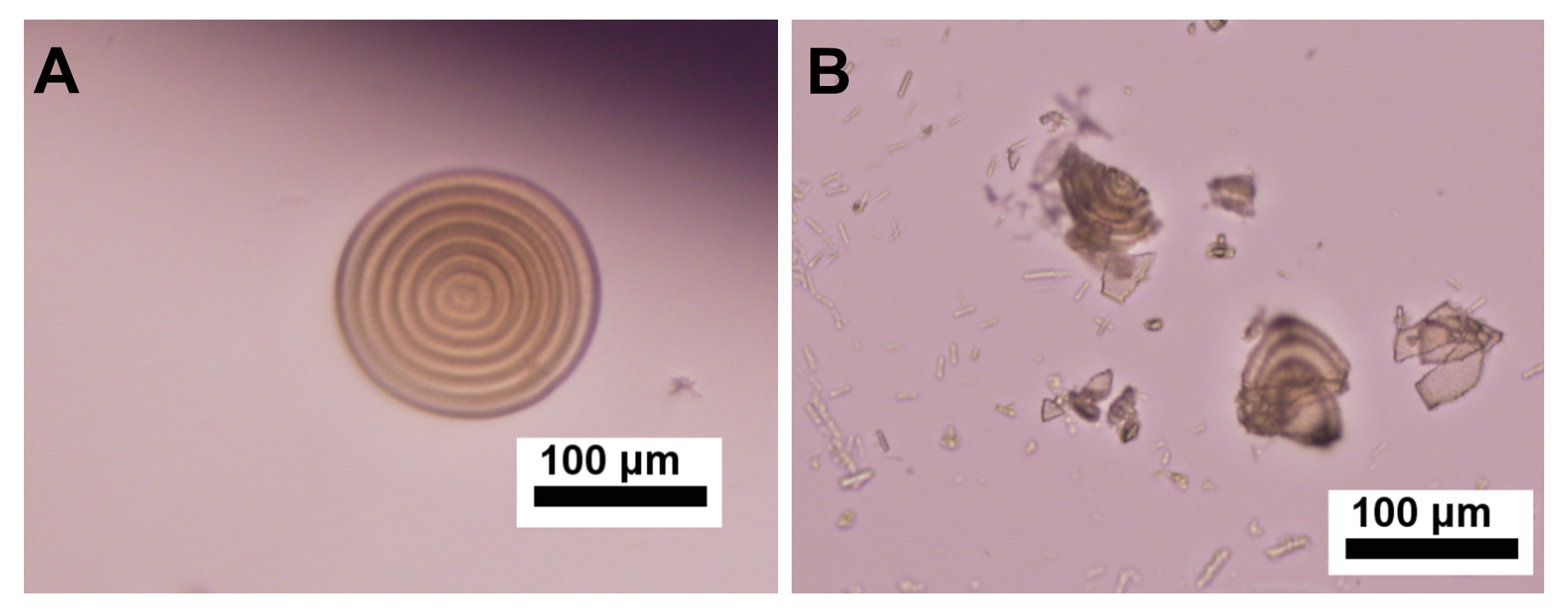

Supplement: Figure S5 — Transmitted light microscopic image exhibiting the dissociated layers of a magnesium carbonate microhemisphere. The microhemisphere was synthesized from a mixture of 120 mM MgAc2, pH 6.5 with 240 mM NaHCO3 (V/V, 1∶1) incubated at T setting of 55°C for 6 h. (A) Intact microhemisphere. (B) Crushed fragments. (TIF) [file pone.0088648.s005.tif]

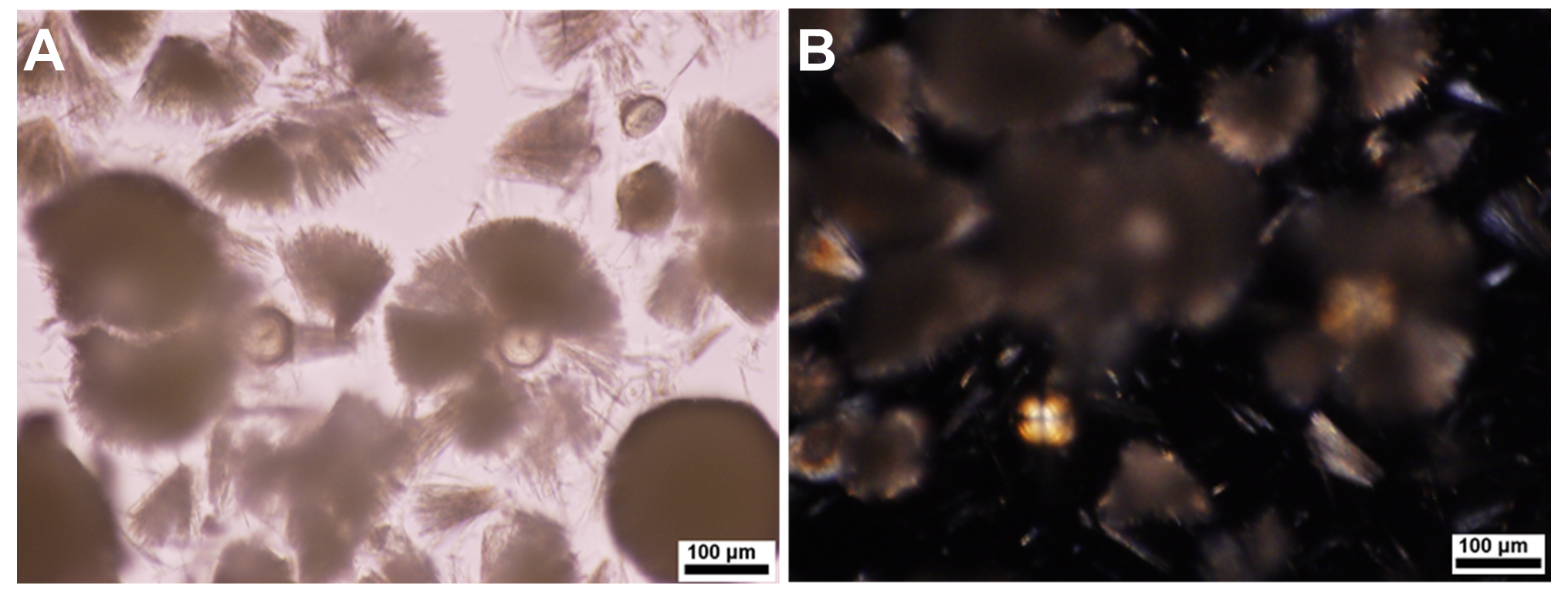

Supplement: Figure S6 — Microspheres synthesized in colloidal suspension of a mineralization system with high initial pH under temperature oscillation condition. The product was from a mixture of 240 mM MgSO4 with 500 mM NaHCO3 of adjusted pH 10.7 (V/V, 1∶1) incubated at T setting of 55°C for 18 h. (A) Transmitted light image showing the structure of fibrous shell and transparent multi-layered core. (B) Cross-polarized light image showing Maltese cross extinction of crystalline and layered core with shell stripped. (TIF) [file pone.0088648.s006.tif]
